# The Role of Attention in Immediate Emotional False Memory Enhancement

**DOI:** 10.1037/emo0000407

**Published:** 2018-06-21

**Authors:** Lauren M. Knott, Mark L. Howe, Enrico Toffalini, Datin Shah, Louise Humphreys

**Affiliations:** 1 Department of Psychology, City University of London; 2 Department of General Psychology, University of Padova; 3 Department of Psychology, City University of London; 4 Department of Psychology, Staffordshire University

**Keywords:** emotion, attention, false memories, DRM paradigm

## Abstract

Two experiments examined the effect of reduced attentional resources on false memory production for emotionally valenced stimuli using the Deese/Roediger-McDermott (DRM) paradigm. Prior research has demonstrated that emotional information is often better remembered than neutral information and that enhanced memory for emotional information is dependent on either automatic or controlled neural processing (Kensinger & Corkin, 2004). Behavioral studies designed to reduce attention resources at encoding have supported neuroimaging findings that indicate high arousal negative stimuli rely more on automatic processing but positive high arousal stimuli rely more on controlled processing. No study has yet examined the attentional resources required to produce emotionally valenced false memories. In Experiment 1, negative, positive, and neutral DRM lists were studied under full or divided attention (DA) conditions, and in Experiment 2, negative and neutral DRM lists were studied under fast (20 ms) or slow (2,000 ms) presentation conditions. Under DA and speeded presentation conditions, higher false memory recognition rates were found for negative compared with positive (Experiment 1) and neutral (Experiments 1 and 2) critical lures. This is the first demonstration of which we are aware that suggests negative false memories are associated with automatic neural processing, whereas positive and nonvalenced neutral false memories are associated with more controlled processing.

Valenced stimuli and emotional events tend to be better remembered than comparable neutral ones (e.g., Bradley, Greenwald, Petry, & Lang, 1992; Cahill & McGaugh, 1995; LaBar & Phelps, 1998; Talmi, Luk, McGarry, & Moscovitch, 2007). This enhanced memory for emotional stimuli appears to be quite a robust and general effect, occurring in the laboratory when tested using both recognition and recall (free and cued) and across a range of stimulus types including pictures, words, and videos (Bradley et al., 1992; Doerksen & Shimamura, 2001; MacKay et al., 2004; Richardson, Strange, & Dolan, 2004; Talmi et al., 2007).

Cognitive theorists attempting to explain the underlying mechanisms for this memorial benefit argue that such emotional stimuli receive more rehearsal or more elaborate processing when encountered than neutral stimuli (e.g., Christianson & Engelberg, 1999) and are likely to trigger personal relevance, which can, in turn, increase performance further on measures of recollection (Kensinger & Corkin, 2003). Concerning the adaptive nature of memory mechanisms, researchers (e.g., Sharot & Phelps, 2004) have also argued that emotional stimuli can be processed by automatic, apparently “preattentive” mechanisms, that facilitate responses toward such meaningful stimuli.

Researchers have suggested that emotion can differ in terms of two underlying dimensions: valence and arousal. Russell (1991) defined valence as varying from pleasant to unpleasant and arousal as varying from calm to excited. Any specific emotion can be conceived of as a pair of values on these continuous scales of valence and arousal. Variation on these two dimensions can cause differences in memory performance such that arousing stimuli, especially those that are negative in valence, lead to better remembering than neutral stimuli (Kensinger, Garoff-Eaton, & Schacter, 2007; Kensinger & Schacter, 2006; McGaugh, 2004).

In support of these behavioral findings, neuroimaging studies have shown that valence and arousal influence memory via modulation of distinct neural mechanisms. Memory performance for arousing (especially negative) stimuli is mediated by the amygdala-hippocampal network. Memory advantages for nonarousing valenced stimuli are due in part to frontally mediated semantic and strategic processes that benefit retention without the key involvement of the amygdala (LaBar & Cabeza, 2006). Studies have also shown that the cognitive processing and neural mechanisms associated with positive stimuli are different than those for negative ones. For instance, Mickley Steinmetz, Addis, and Kensinger (2010) found that the amygdala was activated when processing positive low-arousing stimuli and amygdala efferents weakened as arousal increased for positive stimuli. Electrophysiological evidence has shown that threat-related (high arousing negative) stimuli may elicit earlier encoding (with amplitude enhancement on frontal sites), which means such stimuli could be processed unconsciously in comparison to positive and neutral stimuli (Eimer, Kiss, & Holmes, 2008).

Behavioral research has also supported these conclusions. For example, Talmi et al. (2007) showed that when a concurrent secondary task is used at encoding, attention mediation (i.e., attention is necessary for enhanced effects on memory) accounted for the effect of positive emotion on memory, but not negative emotion (i.e., enhanced effects persist with little attention at encoding). Other studies examining the role of attention, for example by Kensinger and Corkin (2004; also see Kang, Wang, Surina, & Lü, 2014), found this effect to be specific to arousing negative stimuli, with negative nonarousing stimuli and positive (both arousing and nonarousing) stimuli still reliant on controlled processing. Taken together, the growing body of evidence indicates that the cognitive and neurological processing of negative stimuli differs from that of positive and neutral stimuli.

We have seen substantial research investigating the role of emotion on memory. Typically, emotion enhances the amount we remember, but we have also been interested in whether it increases accuracy in what we remember. Over the last 30 years we have seen that memory errors can be associated with emotional stimuli (Neisser & Harsch, 1992; Talarico & Rubin, 2003). In the laboratory, a dominant list learning procedure that has been used to measure the production of so called, spontaneous false memories, is the Deese/Roediger-McDermott (DRM) paradigm (Deese, 1959; Roediger & McDermott, 1995). Here, lists of semantically related words are presented to participants (e.g., *table*, *sit*, *seat*, *couch*, *desk*) but a highly associated word, the critical lure (e.g., *chair*), is missing. At test, participants falsely recall or recognize these critical lures. Moreover, when participants are asked to make remember–know judgments to the critical lures (where a remember response indicates participants can mentally reexperience the presentation of a studied item and a know response indicates participants believe an item is familiar but cannot recollect its presentation) they typically make a remember response (e.g., Roediger & McDermott, 1995).

This procedure has recently been adapted to study false memories for emotional stimuli (e.g., Brainerd, Holliday, Reyna, Yang, & Toglia, 2010; Budson et al., 2006; Howe, 2007; Howe, Candel, Otgaar, Malone, & Wimmer, 2010) where negatively or positively valenced lists (e.g., *harm*, *pain*, *wound*, *punish*, *insult* . . .; critical lure *= hurt* and *hug*, *embrace*, *lips*, *peck*, *affection* . . .; critical lure = *kiss*) are used and compared with neutral lists. Results vary depending on valence and arousal levels of the stimuli, however, a common theme is that when arousal is matched, negative stimuli produce higher false memory rates compared with positive or neutral DRM lists. Thus, the DRM paradigm is a robust measure of vivid false memories and recently, a robust measure of emotional false memories.

There are mainly two opponent theories that explain the production of false memory production. Fuzzy-trace theory (FTT; Brainerd & Reyna, 2005) posits that we store, in parallel, two distinct traces of an item. The verbatim trace represents the surface form of a word (*couch*), whereas the gist trace preserves the meaning (“furniture from a house”). False recollections are based on meaning (gist; *chair* was on the list because I remember items of furniture), especially in the absence of verbatim information. This is often referred to as a dual process approach because recollection of verbatim content suppresses false memories, whereas gist and strong feelings of presence increase false memories. Brainerd, Stein, Silveira, Rohenkohl, and Reyna (2008) hypothesized that emotional content likely increases false memories because negative content increases semantic connections among target events thus increasing gist traces.

Alternatively, theories that are single process driven (true and false memories are attributable to a common process) hypothesize that semantically associated words are stored in a connectionist network (e.g., associative-activation theory [AAT]; Howe, Wimmer, Gagnon, & Plumpton, 2009) and derive from earlier work based on associative memory structures (e.g., Anderson, 1993; Anderson & Bower, 1973; Arndt & Reder, 2003; Collins & Loftus, 1975; Underwood, 1965). As participants view the associative items, activation spreads through the semantic network to related but nonstudied words. According to the activation-monitoring theory (AMT; Roediger & McDermott, 1995), a false memory occurs when participants fail to monitor the source of the activated item, and thus mistakenly believe the critical lure was generated externally (from the study list) rather than internally (from spreading activation). Indeed, any disruptions in source-monitoring have been shown to increase false memories further (see Knott & Dewhurst, 2007). According to spreading activation models, higher false memories associated with negatively valenced compared with neutral DRM lists can be attributed to the well-integrated and dense networks of interrelated concepts for negatively valenced information (e.g., Howe et al., 2009, 2010). In addition, because there are fewer theme nodes associated with many negative than positive or neutral lists, negative critical lures achieve higher levels of activation and are, therefore, more likely to be falsely remembered (Howe & Derbish, 2010; Howe & Wilkinson, 2011; Otgaar, Howe, Brackmann, & Smeets, 2016).

Previous research has also examined the attentional demands required during encoding for the subsequent production of false memory errors using the DRM paradigm. For example, Dewhurst, Barry, Swannell, Holmes, and Bathurst (2007; see also Dewhurst, Barry, & Holmes, 2005; Knott & Dewhurst, 2007) found reduced false recognition rates for critical lures when list items were studied under divided attention conditions. Dewhurst et al. (2007) argued that if the secondary task is sufficient to prevent the generation of associations, critical lure words will not be activated and thus, not falsely recognized as often during test. There are some notable exceptions to this finding where instead, false recall increased after divided attention at encoding. However, in these instances, it is possible that the secondary task was not sufficiently demanding to prevent the generation of associations (Perez-Mata, Read, & Diges, 2002; Otgaar, Peters, & Howe, 2012).

So it appears that for the formation of a false memory, the encoding phase requires a certain amount of attention to allow for the spread of activation (AAT and AMT) or indeed the extraction of the gist trace (FTT). However, given the recent literature surrounding the unique brain activity and automatic processing associated with negative arousing stimuli (Kensinger & Corkin, 2004; Kang et al., 2014), would a task that limited attention during encoding of DRM lists still reduce false memories associated with high arousal, negative valenced stimuli? We would predict that if high arousing negatively valenced stimuli could be automatically processed with reduced attentional resources compared with nonarousing or positively valenced stimuli, then participants should still be able to extract the meaning and activate associative connections when encoding negative high arousing DRM lists under divided attention conditions. Therefore, the purpose of this current study is to examine to what extent attention mediates the enhancement of emotional false memories.

We examined this using two experiments. In Experiment 1, we aimed to replicate the divided attention study by Dewhurst and colleagues (Dewhurst et al., 2005; Knott & Dewhurst, 2007) with the key modification that we included valenced DRM lists. Given the effectiveness of the random number generation task to disrupt attentional resources, we chose this as our divided attention task. Divided attention is designed to limit attentional resources at encoding but participants still have two seconds to encode the stimuli. Individual differences in the ability to carry out the secondary task might mean variation in attentional resources allocated to the encoding task. Thus, similar to Clark-Foos and Marsh (2008), we also aimed to replicate the effect in Experiment 2 using a second procedure to reduce attention at encoding, namely, speeded presentation. This second experiment essentially aimed to replicate a finding by Clark-Foos and Marsh (2008) where fast presentation at encoding reduced overall recognition responses compared with long encoding duration, but negative arousing stimuli were still better remembered compared with neutral nonarousing stimuli. If the enhanced emotional false memory effect for negative valenced items is largely due to automatic processes, then the effect may indeed survive very fast presentation rates.

## Experiment 1

In Experiment 1, we compared neutral DRM lists to both positive and negative DRM lists (both high in arousal). We included positively valenced DRM lists in this first study to examine whether enhanced emotional false memories associated with automatic or controlled processing is mediated by the valence of the stimuli. We also used a between-participants factor for list type and repeated measures for attention. The reason for this was twofold. First, research in the emotional enhanced memory literature suggests a possible distinctiveness effect such that emotional items hold an asymmetrical competition for attention over neutral items (see Talmi et al., 2007; Watts, Buratto, Brotherhood, Barnacle, & Schaefer, 2014). We wanted to eliminate the possibility that distinctiveness of the emotional lists was driving any enhanced false memory effect. Second, Dewhurst et al. (2007) argued that manipulating the attentional task condition between participants could mean that they are able to adjust their decision criteria in what they perceive to be more difficult conditions. Therefore, any effect of the divided attention task could be a criterion effect, rather than an effect of the encoding processes. According to Morrell, Gaitan, and Wixted (2002; see also Wixted & Stretch, 2000), when participants complete a recognition task, with items from both full and divided attention conditions, they are less likely to change their decision criteria during the course of a single test. Although we make no predictions of conservative bias for this study because of the unknown interactions with emotion type, we chose to use repeated measures for attention based on previous findings.

### Method

#### Participants

Ninety-four participants (33 males and 61 females) aged 18 to 46 (*M* = 23.86, *SD* = 7.08) took part in the study and received either course credits or $7 for their participation. A priori power analysis indicated a required total sample size of 90, with a medium effect size and Power (1-β err prob) of 0.95. Informed consent was obtained from all participants and they were fully debriefed at the end of the experiment.

#### Design and stimuli

The experiment followed a 2 (attention: full vs. divided) × 3 (list type: neutral vs. positive vs. negative) mixed factorial design with repeated measures on the first factor. A set of 30 DRM lists (10 positive emotion, 10 negative emotion, and 10 neutral nonemotional lists) were developed using the University of South Florida Free Associations Norms website (Nelson, McEvoy, & Schreiber, 1998). Only words with at least 12 associates were chosen. Each neutral list consisted of 12 associates to the following critical lures: *car*, *chair*, *foot*, *mountain*, *smell*, *window*, *pen*, *shirt*, *high*, and *cup*. Each positive list consisted of 12 associates to the following critical lures: *sleep*, *music*, *sweet*, *soft*, *love*, *beach*, *pretty*, *nice*, *laugh*, and *baby*. Each negative list consisted of 12 associates of the following critical lures: *anger*, *dead*, *cry*, *thief*, *fear*, *lie*, *hate*, *hurt*, *alone*, and *sick*. The overall mean backward associative strength (BAS) values were 0.22 for the neutral condition, 0.24 for the positive condition, and 0.21 for the negative condition. BAS between list items and the critical lure has been shown to be key to the production of false memories (e.g., Roediger, Watson, McDermott, & Gallo, 2001), thus it is important to ensure this is matched across list types. A one-way independent samples analysis of varaiance (ANOVA; using post hoc Bonferroni comparisons, *p* < .05) showed that these conditions did not differ significantly on BAS, *F*(2, 27) = 0.42, *p* = .66.^1^ Available valence and arousal ratings for the list items and critical lures were taken from the Affective Norms for English Words (ANEW; Bradley & Lang, 1999). For list items, there was a significant difference in valence, *F*(2, 27) = 122.45, *p* < .001, where negative lists were significantly lower compared with neutral and positive (both *p*s < .001), and positive lists were significantly higher than negative and neutral lists (both *p*s < .001). There was a significant effect for arousal, *F*(2, 27) = 7.63, *p* = .002, which showed that neutral lists were lower in arousal than positive and negative lists (both *p*s < .05). There was no difference in arousal for positive and negative lists (*p* = .78). For critical lures, the pattern was the same for valence, *F*(2, 24) = 155.68, *p* < .001, where valence was higher for positive, compared with neutral and negative, and neutral was higher than negative (all *p*s < .001). There was also a significant effect for arousal, *F*(2, 24) = 3.67, *p* = .04. There was a difference between negative CLs and neutral CLs (*p* < .05). Importantly there was no difference in arousal ratings for negative and positive lists (*p* = .51).^2^ The means for all negative, positive, and neutral study items and critical lures for Experiment 1 are shown in Table 1.[Table-anchor tbl1]

The order of attention conditions was counterbalanced such that half of the participants in each list type group viewed lists with full attention (FA) followed by divided attention (DA) and the other half of the participants viewed the lists with DA followed by FA. Further, the order of list presentation was randomized for each participant, and each list was seen an equal number of times in FA and DA conditions across participants. Each word was presented on a computer screen using E-prime, shown centrally in black, with 80-point Arial Rounded MT bold font on a white background.

The recognition tests consisted of 60 items: 10 critical lures (one for each of the lists presented at study), 30 target words (three items from each list), and 20 weak and unrelated distractors (10 weakly related and 10 unrelated). Similar to the procedure adopted by Roediger and McDermott (1995), weakly related distractors were chosen from the bottom (or near the bottom) of the associate list from Nelson et al. (1998) but were not presented at encoding. The unrelated distractors were matched for valence depending on the list type condition (i.e., high arousal negative items were chosen for the negative-emotion condition). Each test employed a two-step procedure where participants were required initially to make an old–new response for each item, followed by a remember–know–guess judgment to those items they responded to with an item as old response. The E-prime software (Version 2.0) was used for presentation and data collection.

#### Procedure

Participants were randomly assigned to either a neutral (*n* = 32), positive (*n* = 31), or negative (*n* = 31) list condition. Before the presentation of each list, an on-screen instruction (List 1, List 2, List 3, etc., lasting for 2 s) preceded each list, after which 12 associates appeared individually for 2 s, with each word separated by a 1 second interval. List items were presented from strongest to weakest in associative strength. Half of the lists were subjected to FA and half to DA. For the DA condition, participants engaged in a concurrent task that required them to randomly generate numbers (referred to as RNG for the remainder of this article) between 1 and 20 in time with a metronome in the background every 750 ms. Participants were told to maintain correct speed and correct level of randomness and to avoid counting incrementally or to follow any familiar sequences. The experimenter demonstrated this task before the participant began and consent was gained to record their number generation to allow for a subsequent calculation of the randomness of their output (RNG; Evans, 1978). RNG values range from 0 to 1, with lower values indicating more random sequences. Participants’ number sequences were analyzed using RgCalc, a program designed by Towse and Neil (1998).

After the presentation of all 10 lists, a 10-min distractor task—Sudoku puzzles (with instructions)—preceded the self-paced recognition test. Before the start of the recognition test, participants were told that they were to make an old–new response to each word, followed by an additional recollective experience response (only if the word was labeled as *old*) from a choice of three: remember—if they have a vivid recollection of the word at study (i.e., remembering a specific detail about the word such as an image or thought), know (i.e., if they sense some familiarity of the word being presented at study but lack the conscious recollection of remembering), or guess (i.e., if they were unsure as to whether the word was presented at study or not but lack the confidence to reject it). The responses were made using a mouse click to the corresponding labels that appeared directly underneath each word.

### Results and Discussion

#### Random number generation task

Three participants were removed from all subsequent analyses as they failed to perform adequately on the secondary task. Performance on the random number generation task was compared across emotion conditions to examine any differences in attention devoted to the secondary task. Participants’ number sequences were measured using the RNG score and *N* generated and were analyzed using independent one-way ANOVAs. For RNG scores, there was no significant difference between the three list type conditions (positive = .23, 95% CI [.20, .26], neutral = .20, 95% CI [.17, .23], negative = .23, 95% CI [.21, .26]), *F*(2, 88) = 1.57, *p* = .21, η_*p*_^2^ = .04. For *N* generated, there was also no significant difference between positive (*M* = 105.79, 95% CI [95.47, 116.12]), neutral (*M* = 104.72, 95% CI [91.88, 117.56]), and negative (*M* = 102.93, 95% CI [92.35, 113.52]), *F*(2, 88) = 0.07, *p* = .94, η_*p*_^2^ = .004. Therefore, for this secondary task, there appeared to be no differences in the attentional resources devoted to the completion of the task as a function of list type.

Recognition responses (*old*, *remember, know,* and *guess* judgments) to critical lures, list items, and unrelated fillers were analyzed separately using 2 (attention: FA vs. DA) × 3 (list type: neutral vs. positive vs. negative) mixed factorial ANOVAs with repeated measures on the first factor. Significant interactions were explored using Bonferroni pairwise-comparisons (alpha set at .05). Mean proportions and 95% confidence intervals for the dependent measures are reported in Table 2.[Table-anchor tbl2]

#### Correct recognition

**Figure 1 fig1:**
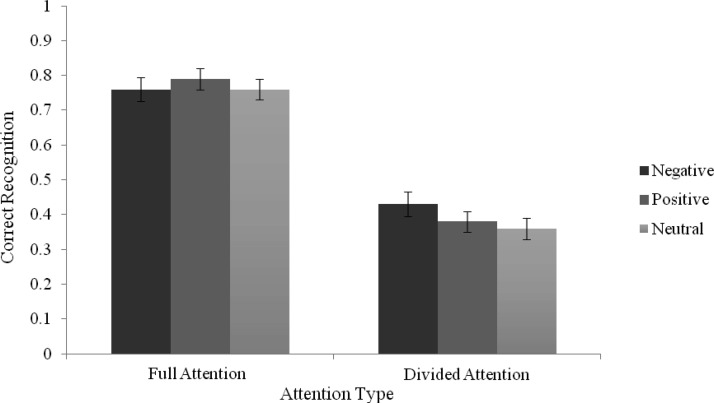
Mean proportions of old responses for the correct recognition of list items as a function of List Type and Attention (Error bars represent standard error) for Experiment 1.

For old responses, there was a significant main effect of attention, *F*(1, 88) = 280.76, *p* < .001, η_*p*_^2^ = .76, where correct recognition was higher in the FA (*M* = .77, 95% CI [.73, .80]) compared with the DA condition (*M* = .39, 95% CI [.35, .43]). There was no significant main effect of list type, *F*(2, 88) = 0.56, *p* = .57, η_*p*_^2^ = .01, or interaction, *F*(2, 88) = 1.07, *p* = .35, η_*p*_^2^ = .02 (see Figure 1). There was a similar pattern for *remember* judgments, with a significant main effect of attention, *F*(1, 88) = 218.31, *p* < .001, η_*p*_^2^ = .71, with higher correct recognition levels for the FA (*M* = .46, 95% CI [.41, .51]) compared with the DA condition (*M* = .09, 95% CI [.07, .11]). There was no main effect of list type or interaction (both *F*s < 1). For know judgments, there was a significant main effect of attention, *F*(1, 88) = 6.23, *p* = .01, η_*p*_^2^ = .07, where again, correct recognition was higher in the FA (*M* = .18, *95% CI* [.15, .21]) compared with the DA condition (*M* = .14, 95% CI [.11, .16]). There was no main effect of list type (*F* < 1, *p* = .51), however there was a significant interaction, *F*(2, 88) = 3.29, *p* < .05, η_*p*_^2^ = .07. Analysis of the simple main effects (SME) using paired samples *t* tests showed no significant difference between attention conditions for either negative, *t*(29) = .08, *p* = .94, *d* = –.07, or positive, *t*(28) = −1.04, *p* = .31, *d* = .32, list type conditions, but false know responses for neutral lists was significantly reduced in the DA compared with the FA condition, *t*(31) = −3.18, *p* = .003, *d* = .75. Analysis of SME using one-way ANOVAs showed that false know responses only within the DA condition differed across the emotion conditions, *F*(2, 88) = 3.35, *p* = .04, η_*p*_^2^ = .07. Multiple comparisons with Bonferroni correction revealed that the responses were higher in the negative (*M* = .18, 95% CI [.13, .22]) compared with the neutral (*M* = .10, 95% CI [.07, .14]) condition (*p* = .04). For guess judgments there was a significant main effect of attention, *F*(1, 88) = 6.48, *p* = .01, η_*p*_^2^ = .07, whereby guess responses to correct items was significantly higher in the DA (*M* = .16, 95% CI [.13, .19]) compared with the FA (*M* = .13, 95% CI [.11, .15]) condition. There was no significant main effect for list type or interaction (*F*s < 1).[Fig-anchor fig1]

#### False recognition of critical lures

**Figure 2 fig2:**
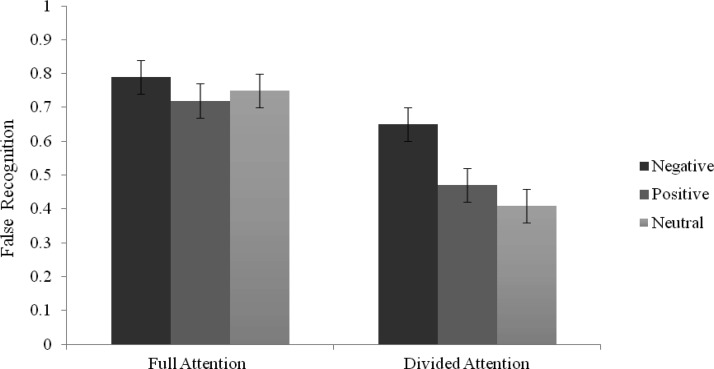
Mean proportions of old responses for the false recognition of critical lures as a function of List Type and Attention (Error bars represent standard error) for Experiment 1.

For old false recognition responses, there was a significant main effect of attention, *F*(1, 88) = 54.57, *p* < .001, η_*p*_^2^ = .38, with higher rates of false recognition in the FA (*M* = .75, 95% CI [.71, .80]) compared with the DA condition (*M* = .51, 95% CI [.45, .57]). There was also a significant main effect of list type, *F*(2, 88) = 4.32, *p* = .02, η_*p*_^2^ = .09, with higher rates of false recognition for negative (*M* = .72, 95% CI [.64, .79]) compared with positive (*M* = .59, 95% CI [.52, .67]) and neutral (*M* = .58, 95% CI [.51, .65]) critical words (*p* = .06 and *p* = .03, respectively). These main effects were qualified by a significant Attention × List Type interaction, *F*(2, 88) = 3.24, *p* = .04, η_*p*_^2^ = .07. Analysis of SMEs using one-way ANOVAs showed that for FA, *F*(2, 88) = .75, *p* = .48, η_*p*_^2^ = .02, there was no significant difference between the three list type conditions (all *p*s > .05). In comparison, for DA, *F*(2, 88) = 5.73, *p* = .005, η_*p*_^2^ = .12, there were higher false recognition rates for negative compared with neutral (*p* = .004) lists, and false recognition rates were marginally higher for negative compared with positive (*p* = .06) lists (see Figure 2). In addition, decomposing the interaction using paired-samples *t* tests between attention conditions for each list type supported the main effect of attention, whereby critical lures were higher in FA compared with DA for positive, *t*(28) = −3.97, *p* < .001, *d* = .86, negative, *t*(29) = −3.34, *p* = .002, *d* = .65, and neutral, *t*(31) = −5.40, *p* < .001, *d* = 1.25 lists. For remember judgments, there was a similar higher false recognition rate in the FA compared with DA conditions, *F*(1, 88) = 43.81, *p* < .001, η_*p*_^2^ = .33. However, there was no main effect of list type, *F* = 2.31, *p* = .11, or interaction, *F* = .02, *p* = .98. For know judgments, there was also a higher rate of false recognition in the FA compared with DA condition, *F*(1, 88) = 9.69, *p* = .003, η_*p*_^2^ = .10, and there was no significant main effect of list type, *F*(1, 88) = 1.85, *p* = .16, η_*p*_^2^ = .04, and an interaction that was approaching significance, *F*(1, 88) = 2.84, *p* = .06, η_*p*_^2^ = .06. The same pattern was observed in know judgments as overall old responses. That is, there were no differences in false recognition rates in the three list types during FA, *F*(2, 88) = 1.06, *p* = .35, η_*p*_^2^ = .02, however negative items produced the highest know judgments to critical lures in the DA condition, *F*(2, 88) = 4.40, *p* = .02, η_*p*_^2^ = .09 (see Table 2). In addition, a significant difference across attention conditions was found only for neutral lists, *t*(31) = −3.17, *p* = .003, *d* = .76, whereby false recognition rates were higher in the FA (*M* = .34, 95% CI [.25, .43]) than the DA (*M* = .17, 95% CI [.11, .23]) condition. Finally, for guess judgments, there were no significant main effects or interaction (all *F*s < 1.50).[Fig-anchor fig2]

#### False recognition of weak-related and unrelated distractors

For weakly related filler items, there was a significant difference in list type for old responses, *F*(2, 88) = 7.69, *p* < .001, η_*p*_^2^ = .15, with higher false recognition rates for the neutral (*M* = .31, 95% CI [.25, .37]) compared with positive (*M* = .19, 95% CI [.12, .26]) and negative (*M* = .13, 95% CI [.07, .20]) items (both *p* < .05), with no significant difference between positive and negative (*p* = .71). There was no significant main effect of attention, *F*(1, 88) = .16, *p* = .69, η_*p*_^2^ = .002, or List Type × Attention interaction, *F*(2, 88) = 2.33, *p* = .10, η_*p*_^2^ = .05. For remember judgments, there were no significant main effects (both *F*s < 1.5, *p* = .14) or interaction, *F*(2, 88) = 1.94, *p* = .15, η_*p*_^2^ = .04. For know judgments, there was a significant main effect for list type, *F*(2, 88) = 3.36, *p* = .04, η_*p*_^2^ = .02, but although Bonferroni pairwise comparisons showed a similar pattern to overall old responses, these differences were not significant between either neutral and positive (*p* = .08) or neutral and negative (*p* = .09) stimuli. Finally, for guess judgments, again there was a significant main effect of list type, *F*(2, 88) = 3.17, *p* < .05, η_*p*_^2^ = .07, but only a significant difference between negative (*M* = .07, *95%* CI [.03, .12]) and neutral items (*M* = .16, 95% CI [.11, .20], *p* = .04). There was no significant main effect of attention, *F*(1, 88) = .001, *p* = .98, η_*p*_^2^ = .00 or List Type × Attention interaction, *F*(2, 88) = 1.54, *p* = .22, η_*p*_^2^ = .03.

Old recognition responses and remember–know–guess judgments for unrelated distractors were analyzed based on list type using one-way independent ANOVAs. Means and 95% confidence intervals are reported in Table 3. For old responses, *F*(2, 88) = 8.25, *p* < .001, η_*p*_^2^ = .16, there were higher false recognition rates for the neutral and positive compared with negative items (both *p*s < .05). This pattern was not observed in remember judgments, *F*(2, 88) = 1.21, *p* = .30, but it was evident in both know, *F*(2, 88) = 3.19, *p* = .05, η_*p*_^2^ = .07, and *guess*, *F*(2, 88) = 5.04, *p* = .01, η_*p*_^2^ = .10, responses.[Table-anchor tbl3]

#### Signal detection analysis

False alarm rates for recognition tests often require a correction for response bias, thus we also include a signal detection analysis. In the following text, we report values of discriminability (*d*′) and bias (*C*) parameters for critical lures (note that better discrimination for critical lures, means that participants are more likely to discriminate the critical lure from the unrelated item) for old responses only.^3^ The results of *d*′ and *C* are summarized in Table 4. Signal detection measures were also analyzed using separate 2 (attention: full vs. divided) × 3 (list type: neutral vs. positive vs. negative) mixed factorial ANOVAs. Similar to the false recognition response data, the main effect of attention was significant, *F*(1, 88) = 51.31, *p* < .001, η_*p*_^2^ = .37, whereby discriminability was better in the FA compared with DA condition. The main effect of list type was also significant, *F*(2, 88) = 19.09, *p* < .001, η_*p*_^2^ = .30, with better memory discrimination for negative compared with positive and neutral lists, with no difference between the latter two. There was a significant Attention × List Type interaction, *F*(2, 88) = 3.39, *p* = .04, η_*p*_^2^ = .07. Analysis of SMEs using one-way ANOVAs (FA: *F*(2, 88) = 8.22, *p* = .001; DA: *F*(2, 88) = 21.16, *p* < .001) both showed better memory discrimination for negative critical lures compared with positive and neutral in the FA and DA conditions (*p* < .05 for both), and no difference between positive and neutral (*p* = 1.00 for DA and *p* = .86 for FA). Analysis of SMEs using paired samples *t* tests to examine discrimination between attention conditions for each list type supported the main effect of attention, with better discrimination in FA across all three list types (all *p*s < .05).[Table-anchor tbl4]

Analysis of the criterion *C* revealed more conservative bias for items encoded in the DA than FA condition, *F*(1, 88) = 51.31, *p* < .001, η_*p*_^2^ = .37. There was no main effect of list type, *F*(1, 88) = 0.81, *p* = .45, η_*p*_^2^ = .02, but there was a significant interaction, *F*(1, 88) = 3.39, *p* = .04, η_*p*_^2^ = .07. However, analysis of the SMEs showed no significant effects other than a trend representing a more conservative bias for negative compared with neutral and positive lists in the FA condition, *F*(2, 88) = 2.73, *p* = .07, with no differences in the DA condition, *F*(2, 88) = 0.36, *p* = .70.

The main aim of this experiment was to examine the role of attention in the production of false memories for emotional and neutral critical lures. As stated in the introduction, if high arousing negatively valenced stimuli could be automatically processed with reduced attention, then participants should still be able to extract the meaning and activate associative connections when encoding negative high arousing stimuli. Divided attention at encoding reduced old responses to critical lures but a significant interaction revealed that false responses were higher for negatively valenced compared neutral critical lures, and marginally higher compared with positively valenced stimuli. Signal detection analysis showed enhanced memory discrimination (more false memories to critical lures and fewer false alarms to unrelated fillers) for negative stimuli compared with neutral and positive stimuli in both encoding conditions. It appears that the secondary task had less influence on the recognition of negative arousing stimuli. False memory rates and, in particular, signal detection analysis indicated that participants were still able to produce false memories for negative stimuli that required fewer attentional resources and more automatic processing. This still allowed the semantic activation of the associative connections, something that was somewhat more impaired for positive and neutral stimuli. Here, we speculate that the more controlled processing required to encode the stimuli was hindered under divided attention conditions. This finding is consistent with prior research showing that enhanced veridical memory for positive (and neutral) stimuli was dependent on full attention during encoding, although this was not the case for negative stimuli (Kang et al., 2014; Talmi et al., 2007). One note that should be made here is that, unlike these findings, our experiment did reveal a reduction from FA to DA in all list types. Although this is slightly at odds with the enhanced emotional effect found in the literature, we are dealing with activation of associates to not-presented items, as opposed to veridical recall of presented items. It is difficult, therefore, to claim that this level of activation during DA will be strong enough to produce the same levels of activation as the FA conditions to produce comparable false memory responses. What we can show is that participants are better able to produce higher levels of false recognition to negative emotional, compared with nonemotional stimuli after DA. This first experiment provides a promising result and is one of the first to demonstrate the role of attention on emotional false memory production in the DRM paradigm. The purpose of Experiment 2 was to enhance the generalizability of this unique finding by attempting to replicate this effect using a second procedure designed to reduce attentional resources at study.

## Experiment 2

In the second experiment we aimed to replicate Experiment 2 of Clark-Foos and Marsh (2008) by shortening the study time. We chose to compare 20 ms and 2,000 ms on the basis of previous research examining false memory production under fast presentation speeds (Seamon, Luo, & Gallo, 1998) using standard neutral DRM lists. The question here is whether false memories associated with negative arousing lists will still be higher than neutral lists with very fast presentation rates? If our conclusions from Experiment 1 are correct, and these effects are largely due to automatic processing of negative emotional stimuli, then we would expect heightened false memories associated with negative emotional stimuli even with limited resources available from such fast presentation rates. We also made two notable methodological changes. First, we only compared negative high arousing DRM lists to neutral DRM lists. This was because there were no noticeable differences in performance between positively valenced lists and neutral lists in Experiment 1 with both stimulus types appearing to rely on more controlled processing for false memory production. Second, list type was treated as a repeated measures factor. Although list type was still blocked, this is more typical in the DRM literature and any individual differences as a result of response to emotional stimuli can be eliminated using this procedure.

### Method

#### Participants

Forty-four participants (34 females and 10 males) aged 18 to 31 (*M* = 24.10, *SD* = 5.02) took part in the study and received either course credits or £5 for their participation. A priori power analysis indicated a required total sample size of 36, with a medium effect size and Power (1-β err prob) of 0.95. All participants gave written informed consent and were fully debriefed at the end of the experiment.

#### Design and stimuli

The experiment followed a 2 (presentation speed: 20 ms vs. 2 s) × 2 (list type: neutral vs. negative) repeated measures design. All participants were presented with 20 word lists in total (10 negative and 10 neutral). The negative and neutral lists were taken from Experiment 1, except for the *anger* list which was replaced with a *devil* DRM list. The negative and neutral lists were matched for BAS (*p* = .65), with negative list items and critical lures significantly higher in arousal (*p* < .001 and *p* = .02, respectively) and lower in valence (*p*s < .001) compared with neutral list items and critical lures.

Full counterbalancing procedures were applied. The order of presentation speed was counterbalanced such that each participant was presented with half the lists (five lists) in each list type condition at a presentation speed of 2 s (slow) and the other half of the lists (five lists) at a speed of 20 ms (fast). The order of list type conditions was also counterbalanced, such that half of the participants began with a negative study–test phase followed by a neutral study–test phase. Furthermore, the order of list-presentation within each presentation speed condition was randomized for each participant. All words were presented at the center of the screen 80-point Arial Rounded MT bold font.

Two recognition tests were created, one for the negative condition and one for the neutral condition. Both tests were constructed in the same fashion and were similar to those used in Experiment 1. Each test consisted of 60 words: 10 critical lures (associated with all the fast and slow lists presented at study during a particular study-test phase), 30 target words (three items from each of the fast and slow lists), 10 weak-related distractors, and 10 unrelated distractors. The weak-related distractors were taken from the bottom of the Nelson et al. (1998) normed lists associated with the critical lures. All distractor items matched valence and arousal measures of the target items. The E-prime studio software (Version 2.0) was used for the presentation of the words and data collection.

#### Procedure

Participants took part in two study-test phases, one with negative lists and one with neutral lists. The order of list type was counterbalanced across participants. The procedure for each study-test phase was the same. Before each list was presented, an on-screen instruction preceded each list (List 1, List 2, List 3, etc.) that lasted for 2 s to regain attention. Thereafter, the 12 associates from each list was presented. The presentation of the lists was broken into two blocks with a 1-min break in-between. The first block consisted of five lists with words presented at a speed of 20 ms (fast), and the second block consisted of five lists with words presented at a speed of 2 s (slow). Full counterbalancing took place, with regard order of speed of presentation, use of lists within each speed condition. Participants were instructed to mentally read and memorize the words and were told to pay very close attention before the fast lists were presented.

After the presentation of all 10 lists, a 5-min distractor task (i.e., Sudoku puzzles) preceded a self-paced recognition test. Participants were given clear verbal instructions on how to complete the recognition task. Similar to Experiment 1, participants were told to categorize each word as either old (i.e., encountered at study) or new followed by remember, know, or guess, if recognized as old*.* This process was repeated for the next list type.

### Results and Discussion

Recognition test responses (*old*, *remember*, *know*, and *guess* judgments) to critical lures, studied items, and weak related fillers were analyzed separately using a 2 (speed of presentation: 20 ms vs. 2 s) × 2 (list type: negative vs. neutral) repeated-measures ANOVA. Any significant interactions were further analyzed using paired-samples *t* tests with Bonferroni corrections (alpha set at .025). Recognition test responses (*old*, *remember*, *know*, and *guess* judgments) to unrelated filler items were analyzed separately based on list type using paired-samples *t* test. Mean proportions and 95% confidence intervals for the dependent measures are reported in Tables 3 and 5.[Table-anchor tbl5]

#### Correct recognition

**Figure 3 fig3:**
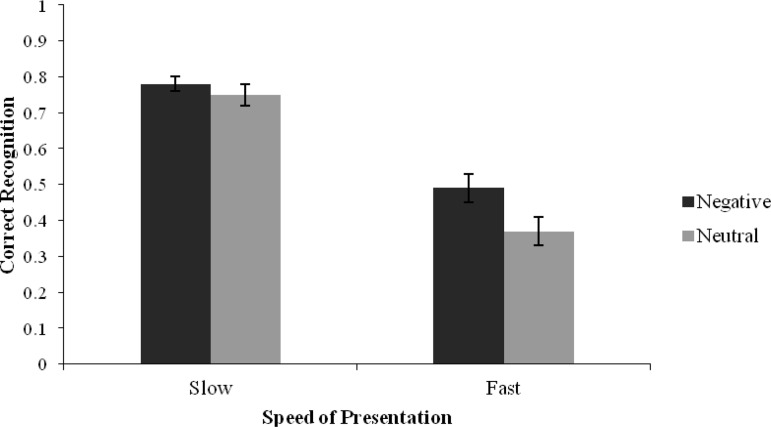
Mean proportions of old responses for the correct recognition of list items as a function of List Type and Presentation Speed (Error bars represent standard error) for Experiment 2.

For *old* responses, there was a significant main effect of presentation speed, *F*(1, 43) = 139.50, *p* < .001, η_*p*_^2^ = .76, whereby correct recognition of studied items was higher for lists that were presented at 2*s* (*M* = .77, 95% CI [.73, .69]) compared with 20 ms (*M* = .43, 95% CI [.37, .49]). There was also a significant main effect of list type, *F*(1, 43) = 10.99, *p* = .002, η_*p*_^2^ = .20, with a higher rate of correct recognition in the negative (*M* = .64, 95% CI [.59, .69]) compared with the neutral (*M* = .56, 95% CI [.51, .61]) condition. However, there was no Presentation Speed × List Type interaction, *F*(1, 43) = 3.62, *p* = .06, η_*p*_^2^ = .08 (see Figure 3). For correct remember judgments, a significant main effect of presentation speed was found, *F*(1, 43) = 126.28, *p* < .001, η_*p*_^2^ = .75, with more remembering of list items in the 2 s (*M* = .45, 95% CI [.39, .50]) compared with the 20 ms (*M* = .14, 95% CI [.10, .17]) presentation condition. There was no significant main effect of list type, *F*(1, 43) = .23, *p* = .64, η_*p*_^2^ = .01, or interaction, *F*(1, 43) = 3.12, *p* = .09, η_*p*_^2^ = .07. For the analysis of correct know judgments, there was a significant main effect of presentation speed, *F*(1, 43) = 10.85, *p* = .002, η_*p*_^2^ = .20, with a similar pattern to correct remember judgments. A main effect of list type was also significant, *F*(1, 43) = 5.05, *p* = .03, η_*p*_^2^ = .11, with more know responses found in the negative compared with the neutral condition (see Table 5). However, no significant Presentation Speed × List Type interaction was found, *F*(1, 43) = .71, *p* = .40, η_*p*_^2^ = .02. For guess judgments, there was a significant main effect of presentation speed, *F*(1, 43) = 8.11, *p* = .007, η_*p*_^2^ = .16, whereby a higher rate of guess judgments was produced for studied words that were presented for 20 ms compared with those presented for 2 s (see Table 5), a reverse pattern to correct remember and know judgments. Guess judgments were also produced more in the negative compared with the neutral condition, *F*(1, 43) = 5.84, *p* = .02, η_*p*_^2^ = .12. The Presentation Speed × List Type interaction, however, did not reach significance, *F*(1, 43) = .002, *p* = .97, η_*p*_^2^ = .00.[Fig-anchor fig3]

#### False recognition of critical lures

**Figure 4 fig4:**
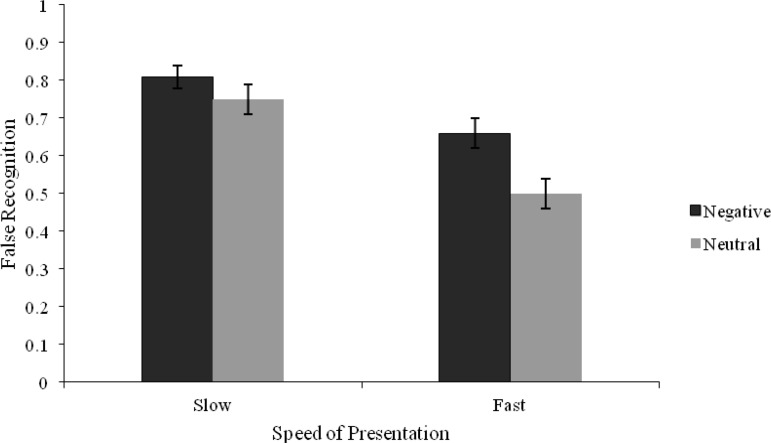
Mean proportions of old responses for the false recognition of critical lures as a function of List Type and Presentation Speed (Error bars represent standard error) for Experiment 2.

For false old responses, there was a significant main effect of presentation speed, *F*(1, 43) = 40.26, *p* < .001, η_*p*_^2^ = .48, with more false responses to critical lures associated with the 2 s (*M* = .78, *95% CI* [.73, .84]) compared with the 20 ms (*M* = .58, 95% CI [.51, .66]) presentation condition. There was also a significant main effect of list type, *F*(1, 43) = 20.17, *p* < .001, η_*p*_^2^ = .32, with negative lures receiving more false memories (*M* = .74, 95% CI [.67, .80]) compared with false memories for neutral lures (*M* = .63, 95% CI [.56, .69]). The main effects were qualified by a Presentation Speed × List Type interaction, *F*(1, 43) = 4.58, *p* = .04, η_*p*_^2^ = .10 (see Figure 4). The simple main effects (SME) of list type revealed no difference in the false recognition of critical lures between negative and neutral conditions when the speed of list presentation was 2 s, *t*(43) = 1.58, *p* = .12, *d* = .25. However, false recognition was higher for negative (*M* = .66, 95% CI [.57, .75]) compared with neutral (*M* = .50, 95% CI [.42, .58]) conditions when lists were studied for 20 ms, *t*(43) = 4.57, *p* < .001, *d* = .58. The SMEs of presentation speed supported the main effect whereby false recognition rates were higher when the presentation rate was 2 s (*M* = .81, 95% CI [.75, .87]) compared with 20 ms (*M* = .66, 95% CI [.57, .75]) in negative lists, *t*(43) = −3.71, *p* < .001, *d* = .55, and higher at a 2 s (*M* = .75, 95% CI [.68, .83]) compared with a 20 ms (*M* = .50, 95% CI [.42, .58]) presentation speed, *t*(43) = −6.09, *p* < .001, *d* = 1.01, for neutral lists. For the analysis of false remember judgments, there was a significant main effect of presentation speed, *F*(1, 43) = 21.54, *p* < .001, η_*p*_^2^ = .33, with higher false remember judgments to critical lures found in the 2 s (*M* = .37, 95% CI [.29, .44]) compared with the 20 ms (*M* = .20, 95% CI [.14, .26]) presentation condition. A significant main effect of list type was also observed, *F*(1, 43) = 4.39, *p* < .05, η_*p*_^2^ = .09, following a similar pattern to old responses. However, there was no Presentation Speed × List Type interaction, *F*(1, 43) = 1.61, *p* = .21, η_*p*_^2^ = .04. For false know judgments, there was a significant main effect of presentation speed, *F*(1, 43) = 9.63, *p* = .003, η_*p*_^2^ = .18, with the direction of the result similar to false old and remember responses. The main effect was also significant for guess judgments, *F*(1, 43) = 5.06, *p* < .05, η_*p*_^2^ = .1, but revealed an opposite pattern. The main effect of list type and the Presentation Speed × List Type interaction for false know and guess judgments did not reach significance (all *F*s < 2, *p*s > .05).[Fig-anchor fig4]

#### False recognition of weak-related and unrelated distractors

For weakly related filler items, there was a significant difference in list type for old responses, *F*(1, 43) = 27.55, *p* < .001, η_*p*_^2^ = .39, with higher false recognition rates for the neutral (*M* = .24, 95% CI [.18, .31]) compared with negative (*M* = .10, 95% CI [.06, .14]). There was no significant main effect of speed of presentation, *F*(1, 43) = .07, *p* = .80, η_*p*_^2^ = .002 or List Type × Speed of Presentation interaction, *F*(1, 43) = 1.23, *p* = .27, η_*p*_^2^ = .03. For remember judgments, there was a similar significant main effect of list type, *F*(1, 43) = 4.22, *p* < .05, η_*p*_^2^ = .09, with higher false recognition rates for the neutral (*M* = .04, 95% CI [.02, .05]) compared with negative (*M* = .01, 95% CI [.001, .03]). There was no significant main effect of speed or interaction (both *F*s < 1). The same pattern was observed for know judgments, with more false alarms to neutral (*M* = .08, 95% CI [.04, .11]) compared with negative (*M* = .04, 95% CI [.03, .06]) weak related fillers, *F*(1, 43) = 6.21, *p* < .05, η_*p*_^2^ = .13, but no significant main effect of speed or interaction (both *Fs* < 1). Similarly with guess judgments, more false alarms were made to neutral (*M* = .13, 95% CI [.10, .17]) compared with negative (*M* = .06, 95% CI [.03, .09]) weak related fillers, *F*(1, 43) = 11.54, *p* = .001, η_*p*_^2^ = .21, but there was no significant main effect of speed or interaction (both *Fs* < 1).

Paired-samples *t* tests were used for the analysis of unrelated fillers (see Table 3). For false old responses to unrelated filler items, false recognition rates did not differ between neutral and negative unrelated items, *t*(43) = −.46, *p* = .65, *r* = .07, and this pattern was further observed in the remember, know, and guess judgments (all *t*s < .50).

#### Signal detection analysis

Similar to Experiment 1, we report values of discriminability (*d*′) and bias (*C*) parameters (see Table 4) for critical lures.^4^ Signal detection measures were also analyzed using separate 2 (Speed of Presentation: 20 ms vs. 2 s) × 2 (list type: negative vs. neutral) repeated-measures ANOVA. There was a main effect of speed, *F*(1, 43) = 41.29, *p* < .001, η_*p*_^2^ = .49, whereby discriminability was better in the 2 s compared with 20-ms presentation condition. The main effect of list type was also significant, *F*(1, 43) = 5.83, *p* < .05, η_*p*_^2^ = .12, with better memory discrimination for negative compared with neutral lists. There was also a significant Speed × List Type interaction, *F*(1, 43) = 4.06, *p* = .05, η_*p*_^2^ = .09. Analysis of SMEs using paired samples *t* tests showed no difference in memory discrimination with 2 s, *t*(43) = 1.05, *p* = .30, *r* = .16, but better discrimination for negative compared with neutral critical lures with 20-ms presentation speed, *t*(43) = 3.11, *p* = .003, *r* = .43.

For the analysis of response bias, *C* was greater for 20 ms compared with 2 s, *F*(1, 43) = 41.29, *p* < .001, η_*p*_^2^ = .49, and for neutral compared with negative items, *F*(1, 43) = 4.56, *p* < .05, η_*p*_^2^ = .10. There was a trend in the interaction, *F*(1, 43) = 4.06, *p* = .05, η_*p*_^2^ = .09. Although *C* was higher for neutral compared with negative in both speed conditions, the analysis of SMEs indicated that this difference was not significant for 2 s, *t*(43) = .92, *p* = .36, *r* = .14, but did reach significance for the 20-ms speed condition, *t*(43) = 2.83, *p* = .007, *r* = .40.

Experiment 2 showed that speeded presentation reduced false recognition rates. Importantly, however, during speeded presentation, false memories for negatively valenced, high arousal stimuli were greater than for neutral stimuli. The signal detection analysis supported these findings, with better memory discrimination (more false memories to CLs than false alarms to unrelated fillers) for negative compared with neutral stimuli. This supports the overall findings from Experiment 1, and supports the conclusion that even with limited attention, high arousing negative stimuli can be encoded and associative false memories can be created. For Experiment 2, a similar effect was observed for veridical recognition, with greater correct recognition responses for negative compared with neutral stimuli in the speeded presentation condition. This supports previous research from the emotion enhanced memory literature (e.g., Clark-Foos & Marsh, 2008; Kensinger & Corkin, 2004; Kang et al., 2014; Talmi et al., 2007), and although this effect was not significant in Experiment 1, the pattern is similar. It may be that the speeded presentation is even more attention limiting than the divided attention condition, where participants may well have still been able to process the semantic links in neutral lists. Research has shown that the effects of organization of list information, that is, lists that are categorically related, can dilute the enhanced memory effect (Talmi et al., 2007). This is often replicated in studies manipulating emotion in DRM lists (e.g., Howe et al., 2010).

## General Discussion

To summarize, these two experiments provide evidence that false memories associated with high arousing negative stimuli require fewer attentional resources and appear to be associated with automatic processing during encoding. In comparison, false memories associated with high arousing positive stimuli (Experiment 1) and nonarousing neutral stimuli (Experiments 1 and 2) are mediated by secondary-task performance requiring attentional resources to successfully encode and activate the nonpresented critical lure. In Experiment 1, this was examined using a concurrent secondary task that divided attention between the encoding task and a random number generation task. In Experiment 2, following a similar paradigm to Clark-Foos and Marsh (2008), attention was limited by reducing the exposure time available during the encoding phase. Both divided attention and fast processing time produced similar results across both studies demonstrating that, at least for the purposes of our studies, both conditions reduce processing and attention to encoding in a comparable manner.

It appears then, as has been evident in the emotion enhanced memory literature, that negative emotional false memories are also associated with automatic processing for negative stimuli. Neurocognitive research suggests that emotion modulates memory through an automatic route primarily consisting of the amygdala and hippocampal brain regions. These areas are considered to be less dependent on the availability of attentional resources (Clark-Foos & Marsh, 2008; Kang et al., 2014; Kern, Libkuman, Otani, & Holmes, 2005; Talmi et al., 2007). Specifically, information that is negatively valenced and highly arousing can be processed automatically and rapidly through this automatic route. This explanation has been supported with neuroimaging studies (Kensinger & Corkin, 2004), but also by previous behavioral studies that have shown an independent attention effect on a veridical memory advantage for negative stimuli (Kern et al., 2005; Talmi et al., 2007). Unlike negative stimuli, memory for positive (and neutral) stimuli is dependent on the intentionality to encode the information and thus, is reliant on more controlled processing. This result supports previous research (Kang et al., 2014) and the suggestion that positive stimuli require more elaborative processing (Fredrickson, 2004) and the work by Mickley Steinmetz et al. (2010) showing that the effect of arousal for positive stimuli is restricted to the amygdala efferents, which weakens as arousal increases. This is unlike the more widespread effect and enhanced connectivity between nodes within the emotional memory network for negative items (Kang et al., 2014). As a side note, this biological difference for valenced stimuli is consistent with an evolutionary perspective on memory and emotion. Limited research has examined the effect of pre- and postgoal emotion on subsequent remembering. That is, we feel negative emotion when goals are threatened and feel positive emotion when goals have been achieved (Levine & Edelstein, 2009). In relation to memory work, research has shown that information associated with uncompleted goals (e.g., negative, threat-related stimuli, high in arousal) tend to be well remembered because they are still needed for survival, whereas information relevant to completed goals that are no longer needed, tend to be forgotten (Förster, Liberman, & Higgins, 2005). This is an interesting and seemingly under researched explanation for the effects of different emotions on memory.

Drawing on theoretical models of false memory production, we can provide an account for the enhanced false memories associated with negative stimuli. Associative-activation theories (e.g., AAT, Howe et al., 2009; AMT, Roediger & McDermott, 1995) propose that for a false memory for a critical lure to occur, the associative links between concepts/nodes need to be activated. Here, more false memories associated with arousing negatively valenced, compared with neutral, DRM lists can be attributed to the denser associative networks containing highly interrelated concepts. As well, because there are fewer theme nodes associated with negatively valenced than neutral information, activation of the negative critical lure is almost a certainty (Howe et al., 2009, 2010; Otgaar et al., 2016). Although FTT (Brainerd et al., 2008) distinguishes between two opponent processes, in a similar manner, they argue that valence (both positive and negative) strengthens gist traces, relative to neutral content, by increasing the semantic connections among target events, but that these connections are more salient for negative compared with positive valence. Although not tested here, they also argue that low or moderate arousal strengthens verbatim traces, but high levels weaken verbatim traces, thus causing an increase in false memories (Bookbinder & Brainerd, 2016).

Both theories can account for the greater false memory rates associated with negative high arousal stimuli in the reduced attention condition. That is, with reduced attentional resources, high-arousing negative stimuli that are automatically processed should allow for the extraction of meaning (or gist) or the activation of associative connections when encoding negative high arousing DRM lists. In comparison, the encoding of positive (and neutral) stimuli requires more elaborate and controlled processing, thus reduced attention hinders successful encoding and reduces the activation of nodes within the positive emotion (and neutral) memory network. Indeed, the neurobiological finding of Mickley Steinmetz et al. (2010) supports this pattern of results and provides an important explanation for the role of valence and arousal in the production of false memories. That is, negative items high in arousal rely on more automatic processing and that arousal only enhances connectivity between other nodes of the emotional memory network for negative items, not positive items. We need to explore this explanation further and examine false memories for positive and negative nonarousing stimuli. However, on the basis of previous emotion enhanced memory research (e.g., Kang et al., 2014), we would predict that processing negative nonarousing stimuli is dependent on the PFC-hippocampal network associated with controlled processing. If true, then we should see a reduction in false recognition of negative, nonarousing critical lures when controlled processing is hindered.

All old responses required a recollective experience judgment. It is worth noting that more old responses were followed by remember judgments for critical lures in the full attention and slow presentation conditions compared with the divided and fast presentation conditions. Moreover, and in line with previous emotional DRM literature (Knott & Thorley, 2014; Ruci, Tomes, & Zelensik, 2009), more remember responses were made to critical lures associated with negative compared with neutral list items. However, there were no interaction effects on remember responses and in fact, for Experiment 1, participants were more likely to associate old responses with a feeling of knowing rather than remembering. We can only speculate why this might be. Although there are limitations with this, the remember/know procedure is the most widely used method to measure the recollective experiences associated with familiarity and recollection (Migo, Mayes, & Montaldi, 2012). According to dual process theories of memory, familiarity is considered to be a rapid, automatic process whereas recollection is a slow, controlled process that reflects the conscious retrieval of contextual details (Jacoby & Dallas, 1981). As familiarity is relatively automatic, any reduction in conscious resources at encoding leaves familiarity as the primary basis for responding (e.g., Jacoby, 1999). Indeed, a number of studies have shown that levels-of-processing manipulations, including divided attention, affect recollection more than familiarity (e.g., Jacoby, Woloshyn, & Kelley, 1989; Khoe, Kroll, Yonelinas, Dobbins, & Knight, 2000). We offer this explanation with some caution, as we would expect a similar pattern in Experiment 2. This may require further investigation, but Clark-Foos and Marsh (2008) argued that rather than a rigid relationship between emotion and recollective/familiarity processing, the reliance on a particular process is likely due to the specifics of the learning episode and the conditions under which memory is tested.

Of note, old responses to filler items were significantly lower for negative compared with neutral items in Experiment 1 with no difference in Experiment 2. There have been mixed findings regarding differences in recognition responses to filler items for emotional versus neutral stimuli in the DRM literature. Previous research has shown either no difference between negative and neutral stimuli (e.g., Dehon, Larøi, & Van der Linden, 2010; El Sharkawy, Groth, Vetter, Beraldi, & Fast, 2008), or greater false alarms to negative compared with neutral (e.g., Budson et al., 2006; Howe et al., 2010). There are no discernable differences in the methodologies used, however, the relatedness of these items to the DRM lists themselves is not made clear and could likely be the cause. Another previously suggested explanation (see Budson et al., 2006; Howe et al., 2010) is that participants adopt a more liberal response bias for emotional stimuli. Although neutral fillers may be weakly related to the list items, they are distinctive from each other. In comparison, weakly related emotional items, by their nature, will be more interrelated with other weak related filler items. In comparison to other neutral filler items, emotional filler items are less distinctive. In Experiment 1, item type was a between-participants factor and in Experiment 2, item type was a repeated-measures factor. We make this comparison because we found a lower liberal response bias for negative compared with neutral and positive list types using a between-participants condition (Experiment 1, in a full attention condition), and the typical higher liberal response bias for negative compared with neutral list types in the repeated measures condition (Experiment 2). Although this particular study focuses on the role of attentional resources for false memory production, differences in response bias for emotional and nonemotional DRM lists is clearly an avenue for additional research.

We conclude with a consideration of the forensic implications of these findings. Research in the emotion enhanced memory field has shown that we are better able to recall and recognize materials that are emotionally salient. More recent research has shown that this enhanced effect for high-arousal negative stimuli could be associated with more automatic processing (Kang et al., 2014). We have now shown the same effect for false memories. Thus, we may well remember emotionally arousing negative events in more detail and possibly regardless of any distracting scenario we encounter, but because of the very nature of how memory processes operate, we will also inevitably produce more false recollections for that event. Thus, the current research may have produced some potentially worrisome findings for the forensic field when memory serves as evidence. Of course, we acknowledge that DRM lists may not be representative of “real-life” forensic situations in which entire autobiographical events may be (mis)remembered (e.g., Pezdek & Lam, 2007), but the DRM paradigm has proven to be a useful tool to understand the mechanisms underlying false memory production. Research has provided evidence that, regardless of which methodology is used, word lists (e.g., Howe et al., 2010) or entire events (e.g., Otgaar, Candel, & Merckelbach, 2008), emotional stimuli are more vulnerable to false memories than neutral stimuli.

## Figures and Tables

**Table 1 tbl1:** Mean Values (Including 95% Confidence Intervals) for List Variables as a Function of Emotional List Type

	Negative lists			Positive lists			Neutral lists		
	95% CI			95% CI			95% CI		
List variables	*M*	Lower limit	Upper limit	*M*	Lower limit	Upper limit	*M*	Lower limit	Upper limit
Valence critical lures	2.25	1.99	2.52	5.95	5.14	6.75	7.85	7.36	8.34
Valence list items	3.10	2.74	3.47	5.34	5.11	5.58	6.96	6.42	7.49
Arousal critical lures	6.12	5.28	6.96	4.57	3.58	5.56	5.36	4.47	6.24
Arousal list items	5.55	4.95	6.14	4.26	3.89	4.63	5.16	4.54	5.78
BAS	.21	.15	.26	.24	.19	.29	.22	.17	.28
List connectivity	1.15	.70	1.61	.88	.57	1.18	.93	.38	1.47
LSA	.25	.21	.30	.27	.24	.30	.28	.22	.33
*Note.* BAS = backward associative strength; LSA = latent semantic analysis.									

**Table 2 tbl2:** Proportionate Mean Values (Including 95% Confidence Intervals) for Recognition Responses to Correct Items, Critical Lure, and Weak Related Lures as a Function of Emotion and Attention at Encoding

	Full attention									Divided attention								
	Negative lists			Positive lists			Neutral lists			Negative lists			Positive lists			Neutral lists		
	95%																		
Response type	*M*	Lower limit	Upper limit	*M*	Lower limit	Upper limit	*M*	Lower limit	Upper limit	*M*	Lower limit	Upper limit	*M*	Lower limit	Upper limit	*M*	Lower limit	Upper limit
Correct recognition																		
Old responses	.76	.69	.83	.79	.73	.84	.75	.70	.81	.43	.36	.49	.37	.29	.46	.36	.29	.43
Remember responses	.45	.37	.53	.50	.42	.58	.43	.34	.52	.08	.05	.11	.10	.06	.14	.09	.05	.13
Know responses	.17	.12	.22	.16	.11	.21	.21	.15	.26	.18	.13	.22	.13	.09	.18	.10	.07	.14
Guess responses	.14	.10	.18	.13	.09	.17	.12	.08	.16	.17	.12	.22	.14	.10	.19	.17	.12	.22
Critical lures																		
Old responses	.79	.73	.85	.72	.63	.80	.75	.66	.84	.65	.55	.74	.47	.35	.59	.41	.30	.51
Remember responses	.32	.22	.42	.32	.22	.42	.24	.15	.33	.15	.09	.21	.13	.08	.19	.06	.02	.11
Know responses	.28	.20	.36	.26	.17	.35	.34	.25	.43	.28	.21	.35	.15	.08	.22	.17	.11	.23
Guess responses	.19	.11	.27	.14	.07	.21	.17	.11	.23	.22	.14	.30	.19	.11	.26	.18	.09	.26
Weak related lures																		
Old responses	.12	.05	.19	.17	.09	.25	.36	.26	.45	.15	.08	.21	.21	.13	.29	.26	.16	.36
Remember responses	.01	–.01	.02	.02	–.003	.04	.06	.00	.11	.01	–.01	.03	.01	–.01	.02	.04	–.01	.08
Know responses	.04	.004	.08	.05	.01	.09	.12	.06	.18	.06	.03	.09	.05	.01	.09	.09	.04	.15
Guess responses	.07	.03	.12	.10	.03	.18	.18	.11	.26	.07	.03	.12	.15	.08	.22	.13	.07	.19

**Table 3 tbl3:** Proportionate Mean Values (Including 95% Confidence Intervals) for Recognition Responses to Unrelated Filler Items for Experiment 1 and Experiment 2

	Negative lists			Neutral lists			Positive lists		
	95% CI								
Response type	*M*	Lower limit	Upper limit	*M*	Lower limit	Upper limit	*M*	Lower limit	Upper limit
	Experiment 1: Divided attention								
Unrelated filler items									
Old responses	.08	.03	.14	.24	.18	.30	.22	.16	.27
Remember	.003	–.01	.02	.01	–.01	.02	.02	.004	.03
Know	.02	–.01	.06	.07	.03	.10	.08	.05	.12
Guess	.06	.01	.10	.15	.11	.20	.13	.09	.18
	Experiment 2: Speeded presentation								
Unrelated filler items									
Old responses	.12	.08	.16	.14	.08	.20			
Remember	.02	.00	.04	.02	.00	.03			
Know	.04	.02	.06	.06	.02	.10			
Guess	.07	.04	.09	.06	.03	.08			

**Table 4 tbl4:** Signal Detection Measures of Discrimination (d’) and Criterion Bias (C) for Correct Items and Critical Lures (CL) for Experiment 1 and 2

	Full attention				Divided attention			
	*d*′		*C*		*d*′		*C’*	
List type	Correct	CL	Correct	CL	Correct	CL	Correct	CL
	Experiment 1							
Neutral	1.53	1.46	.06	.10	.43	.60	.61	.52
Negative	2.10	2.03	.27	.31	1.13	1.70	.76	.47
Positive	1.54	1.25	−.06	.09	.35	.60	.54	.41
	Experiment 2							
	Slow (2,000 ms)				Fast (20 ms)			
Neutral	1.86	1.79	.21	.24	.75	1.14	.76	.57
Negative	1.95	1.93	.17	.17	1.12	1.57	.58	.36

**Table 5 tbl5:** Mean Proportions and 95% Confidence Intervals for Recognition Test Responses to Critical Lures and Correct Items as a Function of List Type and Presentation Speed for Experiment 2

	Fast presentation						Slow presentation					
	Negative lists			Neutral lists			Negative lists			Neutral lists		
	95%											
Response type	*M*	Lower limit	Upper limit	*M*	Lower limit	Upper limit	*M*	Lower limit	Upper limit	*M*	Lower limit	Upper limit
Critical lures												
Old responses	.66	.57	.75	.50	.42	.58	.81	.75	.87	.75	.68	.83
Remember	.25	.17	.34	.14	.08	.20	.38	.29	.47	.35	.26	.44
Know	.21	.16	.26	.19	.13	.25	.31	.25	.38	.26	.18	.35
Guess	.20	.13	.26	.17	.12	.22	.11	.06	.17	.14	.09	.19
Correct items												
Old responses	.49	.42	.57	.37	.30	.44	.78	.74	.83	.75	.70	.81
Remember	.16	.12	.20	.11	.07	.15	.43	.38	.49	.46	.39	.53
Know	.17	.13	.20	.12	.09	.16	.23	.19	.28	.21	.16	.26
Guess	.17	.12	.21	.13	.10	.17	.12	.09	.15	.09	.06	.11
Weak related items												
Old responses	.11	.06	.16	.23	.15	.30	.09	.04	.13	.26	.18	.33
Remember	.02	–.004	.04	.04	.02	.07	.01	–.004	.02	.05	.006	.08
Know	.05	.01	.08	.08	.03	.13	.02	.001	.04	.07	.03	.10
Guess	.05	.02	.08	.12	.07	.16	.06	.03	.10	.15	.09	.20

## Footnotes

1 Research has also highlighted that interitem connectivity (how related items are to each other) can affect the production of false memories in the DRM paradigm (McEvoy, Nelson, & Komatsu, 1999). Thus we calculated interitem connectivity using connectivity matrices, where values were available (see McEvoy et al., 1999). Although connectivity was slightly higher for negative lists (see Table 1), this was not significant, *F*(2, 27) = .56, *p* = .58. At the request of a reviewer we also calculated semantic similarity between list items and CLs (latent semantic analysis [LSA] cosines, Landauer & Dumais, 1997). LSA captures the intercorrelations between words from a large text database, such that the meaning of a word is influenced by the contexts (i.e., neighbors) in which that word occurs. The higher the value the more co-occurrence. There was no significant difference in LSA cosines between negative, neutral and positive lists, *F*(2, 27) = .22, *p* = .80.

2 Note that the variation between 27 and 24 degrees of freedom for list items and critical lures reflects the unavailability of valence and arousal values for the *cry*, *pen*, and *shirt* critical lures.

3 Signal detection analysis calculation method and addition analysis for correct recognition are reported here. For *d*′, larger values equal better memory performance, and for *C*, values greater than 0 represent a conservative bias. The calculation of these measures used the common false alarm rate for unrelated lures, specific to the emotion stimuli type. The standard equation for *d*-prime is *d*′ = *z*(Hit) – *z*(FA), *z* is *z* score. For *C* we use, = −0.5(*z*(Hit) + *z*(FA)). Finally, the Snodgrass and Corwin (1988) correction for signal detection measures was applied. To prevent values of 0 and 1, 0.5 was added to hit and false alarm rates and the corrected score was divided by *N* + 1. For signal detection analysis on correct recognition items, results showed similar patterns to the recognition responses. Importantly, there were no interactions for discrimination measures that were not present in the main analysis, however memory discrimination was higher in the full attention compared with divided attention, *F*(1, 88) = 251.82, *p* < .001, η_*p*_^2^ = .74. For list type, *F*(2, 88) = 3.32, *p* < .05, η_*p*_^2^ = .07, discrimination was also higher for Negative compared with Positive items, (*p* < .05), with no difference between negative and neutral or neutral and positive (both, *p* > .05). Similar to critical lure analysis, criterion *C* revealed more conservative bias for items encoded in divided than for those in full attention conditions, *F*(1, 88) = 251.82, *p* < .001, η_*p*_^2^ = .74. There was a tendency for more liberal responses for positive compared with negative items (*p* < .05), with no other significant comparisons, *F*(1, 88) = 0.86, *p* = .43, η_*p*_^2^ = .02. There was no significant interaction, *F*(1, 88) = 0.86, *p* = .43, η_*p*_^2^ = .02.

4 Signal detection analysis for correct recognition items was also conducted for Experiment 2. Again, results showed a similar pattern to correct recognition responses. Memory discrimination was better for slow compared with fast presentation speeds, *F*(1, 43) = 123.33, *p* < .001, η_*p*_^2^ = .74. Both the main effect of list type and interaction were approaching significance, *F*(1, 43) = 3.94, *p* = .053, η_*p*_^2^ = .08, and, *F*(1, 43) = 3.85, *p* = .06, η_*p*_^2^ = .08. As can be seen from Table 4, there was no difference in discrimination during slow presentation, *t*(43) = .65, *p* = .52, *r* = .10, but it was higher for negative compared with neutral in the speeded presentation condition, *t*(43) = 2.69, *p* = .01, *r* = .38. For the analysis of criterion *C,* there was a more liberal response bias for the slow compared with fast presentation speed, *F*(1, 43) = 123.33, *p* < .001, η_*p*_^2^ = .74. There was no main effect of list type, *F*(1, 43) = 2.59, *p* = .12, η_*p*_^2^ = .06, but there was a trend in the interaction, *F*(1, 43) = 3.85, *p* = .06, η_*p*_^2^ = .08, with analysis of SMEs showing no difference in response bias for list type in the slow speed condition, *t*(43) = .56, *p* = .58, *r* = .09, and, although more conservative in the fast condition, participants were more willing to response old to negative compared with neutral critical lures in the fast speed condition, *t*(43) = 2.13, *p* = .04, *r* = .31.
